# *MuscleX*: data analysis software for fiber diffraction patterns from muscle

**DOI:** 10.1107/S1600577524006167

**Published:** 2024-07-30

**Authors:** Jiranun Jiratrakanvong, Jinjian Shao, Jiaqi Li, Miguel Menendez Alvarez, Xintian Li, Prajwal Das, Grant Nikseresht, Nikhil Miskin, Ran Huo, Jules Nabon, Tristan Leduc, Eric Zhang, Weikang Ma, Gady Agam, Thomas C. Irving

**Affiliations:** a BioCAT, CSRRI and Department of Biology Illinois Institute of Technology Chicago IL60616 USA; b Department of Computer Science Illinois Institute of Technology Chicago IL60616 USA; Australian Synchrotron, Australia

**Keywords:** *MuscleX*, fiber diffraction, data reduction, muscles, fibrous systems, graphical user interfaces

## Abstract

*MuscleX* is an integrated, open-source computer software suite for data reduction of X-ray fiber diffraction patterns from striated muscle and other fibrous systems. It is written in Python and runs on Linux, Microsoft Windows or macOS. Most modules can be run either from a graphical user interface or in a ‘headless mode’ from the command line, suitable for incorporation into beamline control systems. Here, we provide an overview of the general structure of the *MuscleX* software package and describe the specific features of the individual modules as well as examples of applications.

## Abstract 2

*MuscleX* is an integrated, open-source computer software suite for data reduction of X-ray fiber diffraction patterns from striated muscle as well as other fibrous systems.

## 1. Introduction

X-ray fiber diffraction is the method of choice for determining nanometre-scale structural parameters from the sarcomeres in striated muscle *in situ* (Ma & Irving, 2022 ▸). Beamlines at third- and fourth-generation synchrotrons have made it straightforward to collect such information during real physiological experiments in real physiological time, generating many gigabytes of data in a single experimental session. A significant barrier to progress has been the lack of effective software tools to analyze the data. Historically, analysis of diffraction patterns from muscle has been very laborious and time-consuming, representing a significant barrier to timely publication of results. Fiber diffraction practitioners have largely been forced to supplement tools developed decades ago at synchrotron facilities such as *FIT2D* [developed by Hammersley (2016 ▸) at the ESRF] and *BSL* [developed by J. Bordas and G. Mant at the UK Synchrotron Radiation Source (SRS) at Daresbury, UK] with customized ‘home-grown’ software for the specific needs of a given laboratory.

Starting in 1992, there was an attempt to provide a comprehensive and coherent set of software tools for analyzing fiber diffraction patterns in the context of the *CCP13* project. These programs (Squire *et al.*, 2003 ▸) originally ran on various UNIX or UNIX-like platforms and, though useful in expert hands, were not easy to use. A more user-friendly version of the *CCP13* software suite was developed in the form of a single integrated program called *FibreFix* (Rajkumar *et al.*, 2007 ▸) that initially ran on Microsoft Windows platforms and later on various platforms as a Java version distributed as a compiled .jar archive. Both the original *CCP13* code and *Fibrefix* worked well for analyzing the diffraction spots on layer lines in muscle patterns from quasi-crystalline systems such as fish and insect flight muscle, but were not as useful for other common tasks, notably the analysis of the equator of muscle patterns. The most serious deficiency of the *CCP13* based packages, however, was that both the original *CCP13* Unix-based code and *Fibrefix* were conceived as closed-source programs. This is also the case with *Fit2D*. It was not possible to maintain the code once the funding ran out and the developers moved on. Importantly, it was also not possible to adapt the code for situations not anticipated by the original developers.

Here, we describe the new open-source software package *MuscleX* that is optimized for analyzing large sets of X-ray diffraction images. As its name suggests, it was written primarily with the needs of muscle diffraction in mind, but it is expected to be useful for analyzing diffraction patterns from other fibrous systems as well. It incorporates some of the functionality of the *CCP13* package, including the ability to quadrant fold and globally subtract the diffuse background in 2D, as well as newly developed tools for analyzing the meridian of the pattern and individual layer lines. An important capability is an optimized tool for analyzing the equatorial patterns from muscle. The programs are designed to process entire directories of images with minimal user intervention to improve reproducibility, reduce the effects of operator bias and increase efficiency. Analysis tasks that took many weeks can now be done in a matter of hours. The package currently consists of ‘*X-ray Viewer*’, a fast and convenient viewer of sequences of X-ray patterns and associated metadata; ‘*Quadrant Folding*’, to average quadrants of a fiber pattern with optional global 2D background subtraction; ‘*Equator*’ for analysis of equatorial patterns from muscle; ‘*Projection Traces*’ for analysis of 1D intensity projections; and ‘*Add Intensities*’, to combine multiple images in various ways to improve the signal-to-noise ratio (SNR). The *MuscleX* distribution also includes a ‘*Diffraction Imaging*’ module for analyzing scanning diffraction imaging data (https://musclex.readthedocs.io/en/latest/AppSuite/ScanningDiffraction/index.html#scanning-diffraction). A paper describing this package and its capabilities will appear elsewhere. Here, we present an overview of the general structure of the *MuscleX* software package, describe the specific features of the individual components and provide some examples of applications. We conclude with a discussion of current limitations of *MuscleX* and indicate some future directions.

## 2. Description of *MuscleX*

### 2.1. General features

All programs are written in Python and, as such, can be run on many platforms including Linux, Mac OSX and Microsoft Windows. The graphical user interface (GUI) is very user friendly. Initial estimates of parameters are made without any user intervention but can be adjusted as needed. The programs can input all image data types supported by the ‘FabIO’ library (Knudson *et al.*, 2013 ▸) covering most commonly used, commercially available X-ray detectors, with tiff and hdf5 files being the most extensively tested by us. All internal calculations use 32 bit floating point arithmetic and any resulting images exported as 32 bit floating point tiff files. *Matplotlib* (Hunter, 2007 ▸) with *PyQT5* (https://pypi.org/project/PyQt5/) is used for graphical output. The *pyFAI* package developed at the ESRF (Ashiotis *et al.*, 2015 ▸) is used for accelerated radial integration and image calibration. The *lmfit* package (https://lmfit.github.io/lmfit-py/) is used for peak fitting and the *pyMca5* package (Solé *et al.*, 2007 ▸) is used for masking. Background-subtraction codes, originally written in Fortran, from the legacy *CCP13* source code archive https://github.com/scattering-central/CCP13), have been re-implemented in Python with Python-native libraries.

### 2.2. Features common to all routines

Common features to all routines include calibration, instrumental background-subtraction image (empty cell image) and masking. When an image is selected, the calibration settings window will pop up, allowing input of a calibration pattern (*e.g.* from silver behenate) to be input. This allows calculation of an accurate beam center and the conversion from pixels to inverse nanometres. If there is no calibration image or the calibration parameters (wavelength, sample-to-detector distance, beam center) have not been manually set, the image will be processed automatically with the default parameters. The calibration parameters include wavelength, sample-to-detector distance and beam center. When an instrumental background-subtraction image or a mask image is selected, the original image will be subtracted by the instrumental background image and the mask will be applied to mask out regions before subsequent analysis.

### 2.3. ‘Headless’ mode

It is possible to run many of the routines (currently *Quadrant Folding*, *Equator* and *Projection Traces*) from the command line or as part of a script in a special ‘headless’ mode that bypasses the GUI. This allows for automating processing steps. Any necessary parameters can be set by running the interactive GUI version of the program on the first file of a series, setting any necessary parameters, then select save the current settings in a settings file in json format. It is possible to manually edit the settings file as needed to modify any desired parameters. If a settings parameter does not exist, default parameters will be used. The headless mode can process several images concurrently using multiple CPU cores. For example, with a 12-core computer, 12 images can be processed in parallel at the same time, and the results are saved in the same output file. This can be particularly advantageous with time-resolved data. The ‘headless mode’ also allows simple incorporation of the routines into beamline control software or data processing pipelines.

### 2.4. Module descriptions

#### 2.4.1. X-ray Viewer

**Figure 1:**
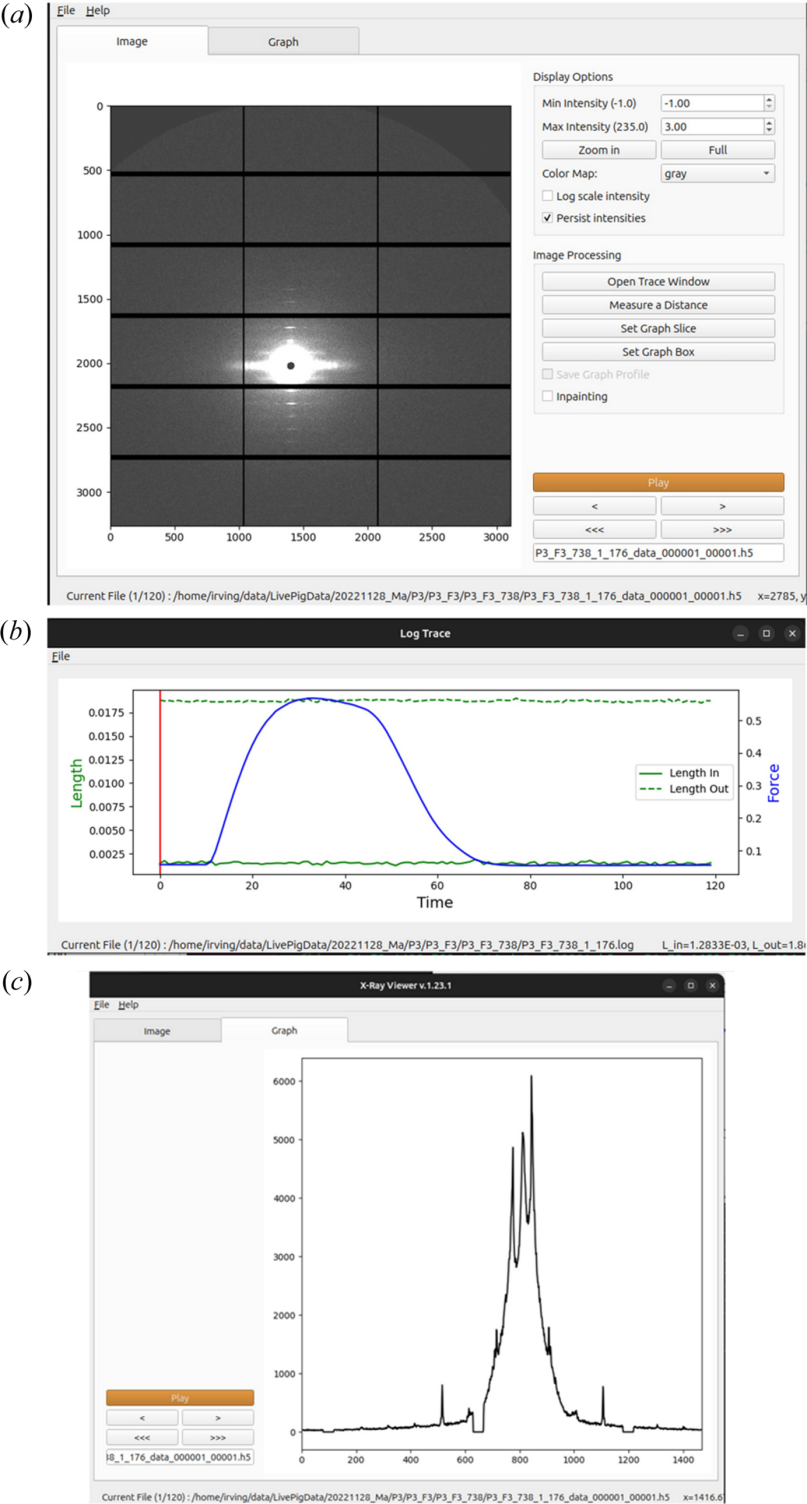
*X-ray Viewer* module of *MuscleX*. (*a*) Main window. (*b*) Force and length changes from the user-supplied log file displaced in the trace window. The vertical red line indicates the frame number currently being viewed. (*c*) Integrated intensity projection along the meridian of the pattern generated by the *Graph Box* tool.

*X-ray Viewer* (Fig. 1 ▸) is designed to display folders containing sequences of images stored as individual tiff images or sequences of individual images stored in hdf5 files, and is able to play these sequences of images as a video. This can be particularly valuable during an experiment where it can be used to quickly observe the evolution of the diffraction data with time to ensure that the experiment is proceeding properly. AS an option, it is possible to make a line selection or a box containing a diffraction feature of interest and display the integrated intensity within the selection as a graph in real time with the ability to save the data as ASCII comma separated variable files for import into other programs. Another feature allows for the display of metadata (*e.g.* muscle force and length data) at the same time as viewing the images recorded during the experiment. To support this display, the metadata needs to be prepared in an ASCII file using a suitable format. An additional tool allows the user to measure the distances (in pixels) between diffraction features in individual images.

#### 2.4.2. Quadrant Folding

**Figure 2:**
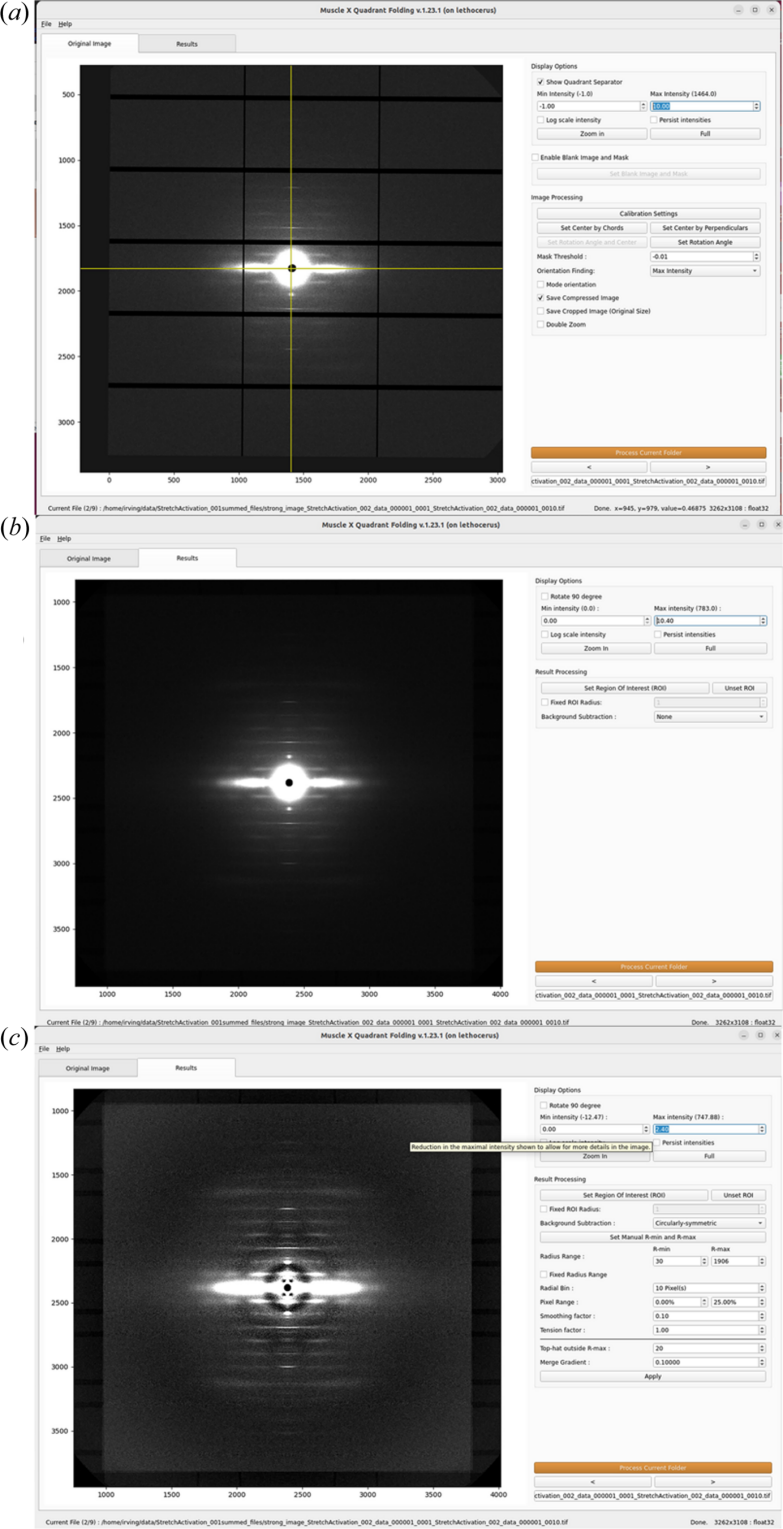
*Quadrant Folding* module of *MuscleX*. (*a*) Main window with setup options. (*b*) Quadrant-folded image. (*c*) Quadrant-folded image with a circularly symmetric background subtracted.

The equator and the meridian of a fiber diffraction pattern separate the pattern into four quadrants. Because of Friedel’s Law, each of the four quadrants will contain the same information. Adding the four quadrants together effectively increases the signal strength by a factor of 4, thus improving the SNR by a factor of 2 (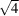). A full diffraction pattern can then be generated by replicating the summed quadrant. Analyzing quadrant-folded images is preferable in many instances due to the improved SNR, which makes fitting the data easier as well as allowing for better diffuse background estimates. The *Quadrant Folding* module (Fig. 2 ▸) is a program for generating such a quadrant-folded image. Another advantage of quadrant folding is that it can compensate for detector imperfections by substituting data from the unaffected quadrants for the affected area. This is particularly useful for data collected using the popular Pilatus (Broennimann *et al.*, 2006 ▸) and EIGER pixel array detectors (Johnson *et al.*, 2014 ▸) from Dectris Inc. that have substantial gaps between the active detection elements. With careful selection of the beam center position, it is often possible to compensate for the gaps in the quadrant-folded image. Once set up, the program can process an entire directory of images without user intervention, either directly from the GUI or in ‘headless’ mode. When an image is selected, the program immediately processes it and saves the quadrant-folded image along with estimated backgrounds (if selected) in a subdirectory of the directory containing the original image.

To process an image, the *Quadrant Folding* program performs several steps as follows: identify the pattern center and rotation angle, define axis-aligned quadrants using the pattern center and rotation angle, flip all quadrants to have the same orientation, average pixels in the four quadrants and replicate the quadrants to form a complete image. A user-selected intensity threshold (‘mask threshold’) is used to exclude from averaging pixels with intensities below the threshold. This allows for exclusion of pixels in detector gap lines from being averaged. The full 2D pattern is then generated by rotations of this image and saved to the results directory.

Fiber diffraction patterns from muscle have a substantial background arising from non-crystalline parts of the muscle that do not diffract coherently, including membranous structures such as sarcoplasmic reticulum and mitochondria, as well as diffuse scattering from the myosin heads. Intact cardiac muscle in particular has a very strong background from the higher amount of connective tissue than in skeletal muscle. This background pattern may have a complex structure, making it difficult to model and effectively remove. Various algorithms have been proposed to attempt to remove this background. No one technique proposed so far can effectively remove the background in all parts of the image without over-subtraction in some parts of the pattern and under-subtraction in others, but some techniques work better than others depending on the type of muscle and the specific preparation. *Quadrant Folding* implements several different algorithms that can be used individually or in combination to produce background-subtracted images for display or subsequent analysis.

There are several options for background subtraction: circularly symmetric, Gaussian smoothing (Ivanova & Makowski, 1998 ▸), roving window (attributed to Dr Paul Langan), smoothed boxcar from the *CCP13* legacy code archived by Diamond Light Source (https://github.com/scattering-central/CCP13) and a new algorithm based on the ‘white top hat’ filtering routine from the *Scikit-Image* Python package (van der Walt *et al.*, 2014 ▸). It is possible to merge different background estimates from the above algorithms by applying them separately at low and high radii.

#### 2.4.3. Equator

**Figure 3:**
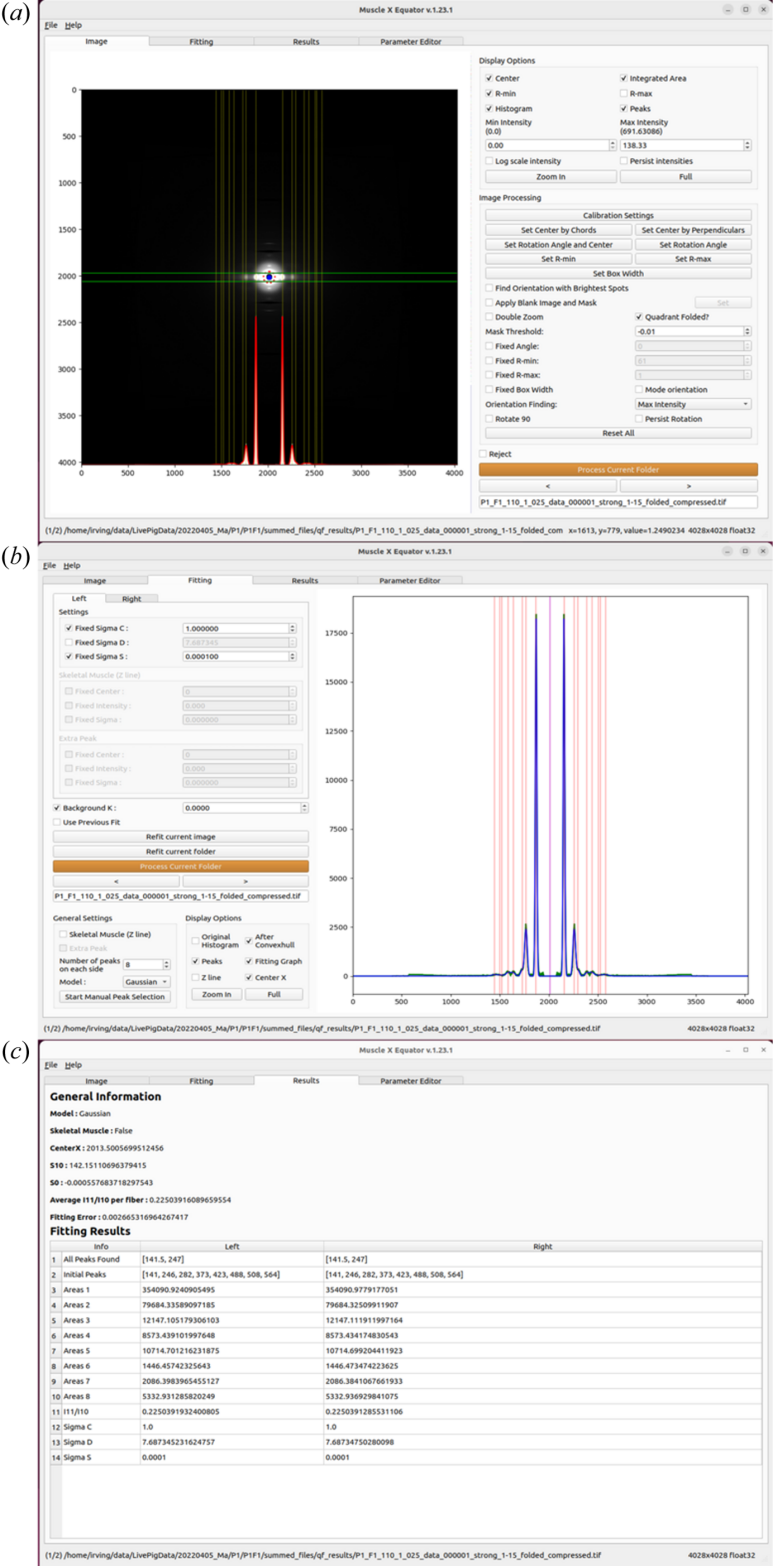
*Equator* module of *MuscleX*. (*a*) Main window with the diffraction image, intensity projection along the equator and the fit. (*b*) Fit window where the user inputs peak parameters for the module along with a detailed view of the fit. (*c*) Results window.

The purpose of the *Equator* program (Fig. 3 ▸) is to analyze the equatorial portion of muscle X-ray diffraction patterns, but it can be used with any fibrous system with hexagonally packed repeating units. Though it is possible for some fibrous systems to have non-hexagonal lattices, all muscle patterns studied to date have had, at least approximately, hexagonal symmetry for the filament lattice, so the restriction to hexagonal symmetry is not a serious limitation.

The *Equator* program is designed to (1) determine the inter-filament lattice spacing, *d*_10_; (2) fit Voigt or Gaussian model functions to a selected number of diffraction peaks in order to estimate their integrated intensities; (3) determine the ratio of the intensities of the 1,1 and 1,0 equatorial reflections (*i.e.* the *I*_11_/*I*_10_ intensity ratio); (4) obtain estimates for parameters reflecting the width of lattice spacing distributions and the degree of liquid-like disorder from the peak widths, assuming an ideally para-crystalline model for the hexagonally packed myofilaments (Ma & Irving, 2022 ▸). Prior to analysis, the center of the pattern and spacing calibration may be calculated from a user-supplied calibration pattern (*e.g.* from silver behenate). In the absence of such patterns, the center is estimated directly from the pattern and the reflection spacings are reported in pixels. The orientation is estimated using one of several algorithms selected by the user and employed to rotate the pattern so that the equatorial axis is horizontal. An integration box is estimated from the reflection widths and used to generate a 1D intensity projection onto the horizontal axis. The diffuse background is subtracted using a convex hull algorithm (Preparata & Hong, 1977 ▸). The diffraction peaks are fitted using the user-defined model (either Gaussian or Voigt functions are currently supported and other peak shape models will be added in a future release) with positions constrained to lie on a hexagonal lattice and the peak widths by the para-crystalline model function. Complex X-ray patterns can be fit using a flexible parameter editor to individually adjust parameters for individual peaks and introduce additional, non-indexing peaks as necessary. The program performs these tasks with as little user intervention as possible in order to improve reproducibility, reduce the effects of operator bias and increase efficiency. The routine can operate on a whole directory of images, either directly from the GUI or in ‘headless mode’, producing results in hours instead of the many weeks previously required for manual processing. Given that not all patterns are amenable to this automated approach, any failed cases are flagged for manual processing within the equator program. Such cases can be processed by manually adjusting the initial parameter estimates and re-running the fits. The results of all fits are saved in a comma separated variable (.csv) ASCII file suitable for reading by spreadsheet programs.

#### 2.4.4. Projection Traces

**Figure 4:**
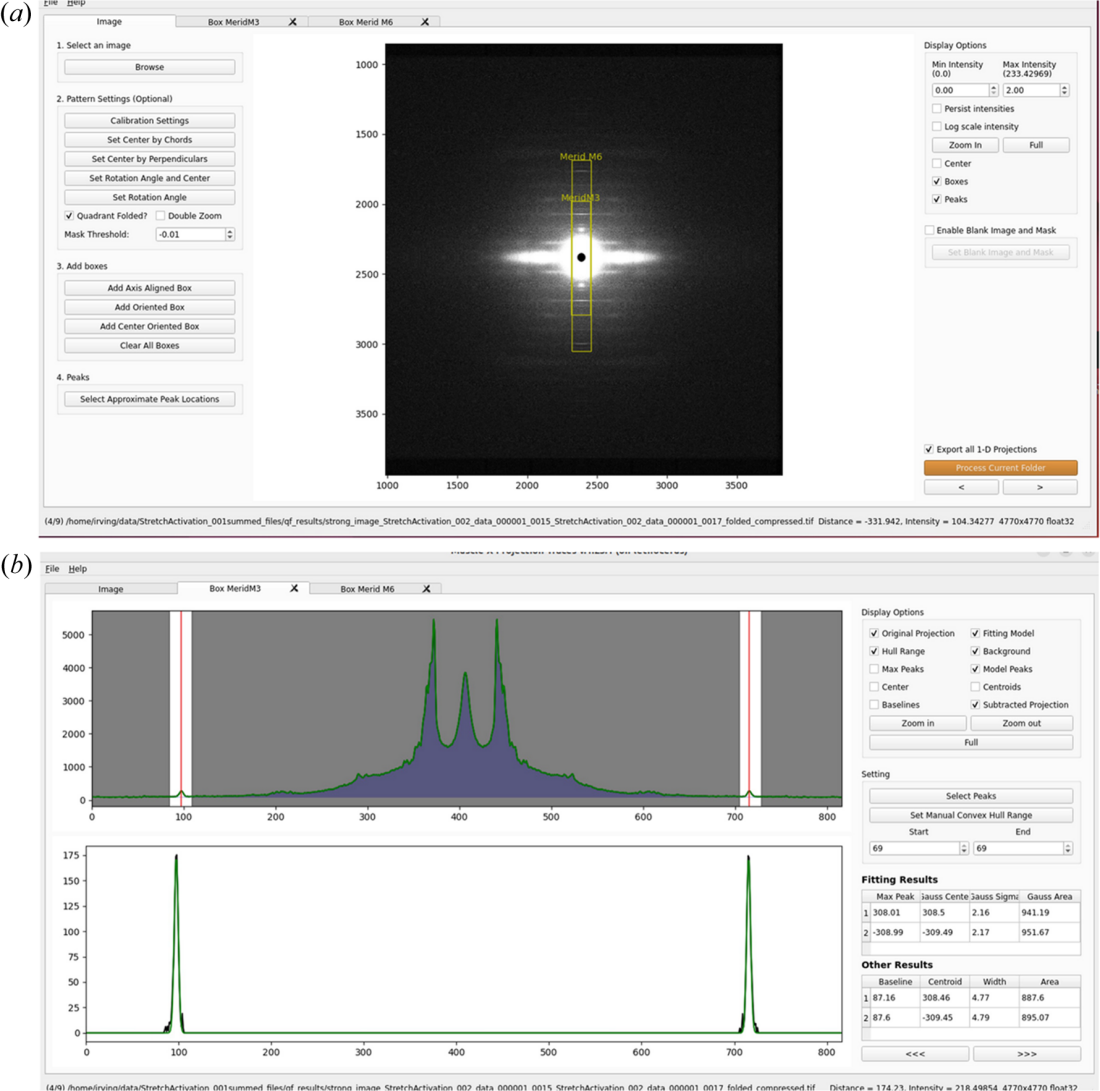
*Projection Traces* module of *MuscleX*. (*a*) Main window where users choose boxed regions to generate integrated intensity projections along with the positions of the features being measured. (*b*) Fit window where the parameters for the fits are selected and the results displayed.

It is usually necessary to reduce 2D diffraction patterns to 1D projections in order to extract accurate position and intensity information from diffraction peaks. It is often helpful but not always necessary to start with background-subtracted images using one of the algorithms implemented in *Quadrant Folding*. The *Projection Traces* program (Fig. 4 ▸) can calculate projections of the integrated intensities aligned either along or parallel to the meridian or the equator as well as at arbitrary angles that need not go through the center of the pattern. The resulting projections can be optionally saved as 1D intensity traces to ASCII files for analysis with other, user-supplied programs. In *Projection Traces*, the user provides initial estimates of the positions of the peaks the user wishes to analyze. If the boxes and peaks are specified for one image in a folder, all other images in the same folder will be processed using these boxes and initial peak positions. Once the program is set up for the desired analysis, it is possible to process whole directories of images without user intervention.

One use of *Projection Traces* is to rapidly and accurately measure the spacings of user-specified meridional reflections as well as peak positions from axial profiles of the layer lines in a series of diffraction images such as those from a time-resolved experiment. The background under the peaks can be modeled either as sums of Gaussian functions or estimated and removed using a convex hull algorithm (Preparata & Hong, 1977 ▸). The integrated intensity under a diffraction peak is modeled as the area of a Gaussian function. The center of the Gaussian function is reported as one estimate of the peak position. An alternative estimate of the peak position that is less sensitive to noise, following Huxley *et al.* (1994 ▸) uses the centroid of the top half of diffraction peak as an unbiased measure. The centroid is calculated as the sum of distances from center weighted by projected intensity in the range between the right and left boundaries on the baseline. This quantity is then divided by the sum of the projected intensity in the peak above the baseline to yield the centroid. In this process, peak width and height are also estimated, allowing the calculation of an integrated intensity using a triangular approximation that can be compared to that estimated by the Gaussian fits. Because of the possibility of changes in peak shape during an experiment, the centroid can be a more objective measure of small changes in the spacing of a diffraction peak, while the area of the Gaussian fit can be a better objective measure of the diffracted intensity. Once the desired peaks and approximate locations have been specified by the operator, an entire directory of images can be processed without user-intervention, either from the GUI or in ‘headless’ mode. Results are recorded in a .csv format data file along with the integrated intensity of the layer line peaks.

#### 2.4.5. Add Intensities

The *Add Intensities* module comprises two programs that are designed to be used with series of images with sequential file names taken in a time-resolved experiment. By adding equivalent images together, it is possible to improve the SNR and observe weaker diffraction features. The first version of the program is ‘*Add Intensities Single Experiment*’ (*AISE*), which is designed to add images from quasi-static conditions during a physiological protocol such as rest and plateau of isometric contraction. It averages user-selected images in an input directory and saves the new image as a tiff file in an output directory. The resulting tiff files are given self-descriptive file names indicating which operation was performed to produce them. These files can then be further processed as needed. A user-supplied parameter controls the number of subsequent images that are added. This effectively results in re-binning of images in time that is applied to the input directory without user intervention. It is also possible to adjust the center and orientations of individual images as necessary as the routine proceeds. *AISE* can also be used to sum user-selected frames from quasi-static regions of a protocol (*e.g.* during the rest period prior to contraction or during the plateau phase of an isometric contraction) and save these to new files in the output directory. The second routine is ‘*Add Intensities Multiple Experiments*’ (*AIME*), which is designed to be used with series of images in multiple folders (or individual hdf5 files), each corresponding to a single experiment. It calculates the sum between images with the same frame number (*i.e.* the same point in a time series) in different directories and saves the resulting summed images as tiff files in a folder in the selected directory. Again, the resulting tiff files are given self-descriptive file names to indicate which operation was performed to produce them. With both versions of the program, it is possible to average instead of summing, as well as observe the individual images prior to being summed, so that any necessary adjustments can be performed.

### 2.5. Workflow

**Figure 5:**
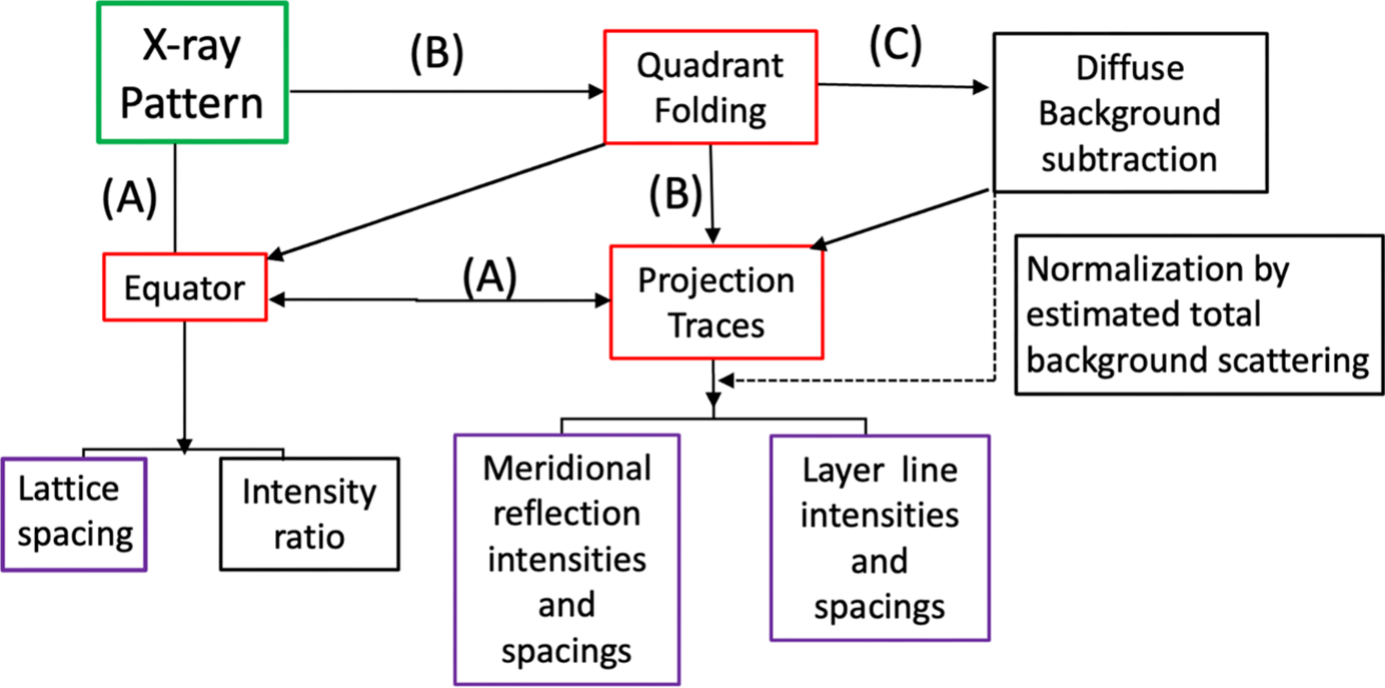
Possible workflows when using the software suite. Path A goes directly from the diffraction pattern to either the *Equator* or the *Projection Traces* modules for analysis of the equatorial and off-meridional diffraction patterns. Path B introduces *Quadrant Folding* as the first step of the analysis prior to *Equator* or *Projection Traces*. Path C adds an additional step after quadrant which estimates the diffuse background intensity distribution that is then subtracted from the diffraction pattern prior to analysis with *Projection Traces*. *MuscleX* modules are indicated by red boxes, other operations by black boxes and output parameters by purple boxes. Note that global background subtraction is usually not necessary or helpful prior to analysis with *Equator*. Note that it is possible to use the total intensity in the estimated background for scaling data from different patterns so they can be compared.

Fig. 5 ▸ shows some possible workflows when using the software suite. With strong diffraction patterns from detectors that do not have gaps between active detector modules, including most CCD detectors, it is possible to go directly to the equator or projection trace modules (Path A) to analyze the equator, meridional and layer line data directly. With most pixel array detectors, it is often desirable to quadrant fold the image to remove the detector gaps and increase the SNR prior to analysis (Path B). In many cases, it is desirable to attempt to estimate and remove the diffuse background from the patterns using the routines built into *Quadrant Folding* prior to analysis with *Projection Traces* (Path C). An additional feature of this mode is an estimation of the total intensity in the estimated background which can be used for scaling.

## 3. Current limitations and future directions

We plan several improvements in four different aspects of the *MuscleX* programs. First, while the cylindrically symmetric background-removal routine typically does an adequate job for display purposes, none of the global background-subtraction routines included in *Quadrant Folding* provide a completely satisfactory background removal on their own. We are currently working on improved global background-subtraction schemes using deep learning (Aranguren Carmona *et al.*, 2020 ▸). Second, analysis of the meridian of the muscle fiber diffraction patterns does not currently consider any substructure within the primary meridional reflections (M3, M6, troponin reflections *etc.*). It also does not attempt to resolve the individual peaks within clusters of peaks around the M1 and M2 forbidden meridional reflections. While knowing the overall intensity and position of the M3, M6 and troponin reflections suffice for many purposes, more detailed analysis relies on exporting the integrated intensity traces to a user-supplied peak fitting program. We are currently developing machine-learning approaches along with time and space deconvolution algorithms to perform global fits to all the meridional reflections in a pattern and to extract more information from poorly resolved peaks. Third, we plan to port additional components of the *CCP13* suite directed towards obtaining integrated intensities of fiber diffraction peaks from fiber diffraction patterns from crystalline muscle systems, such as those of insect flight muscle and bony fish (Squire *et al.*, 2003 ▸). Finally, we are also working on ways to better support the capture and curation of metadata through data analysis chains, in concert with support for the NeXus data format (Könnecke *et al.*, 2015 ▸). Given the increasing quantity of NeXuS format muscle diffraction data obtained on Beamline I22 at Diamond Light Source, full support for images and metadata stored in NeXuS format files will be added to *MuscleX* in a future release.

## 4. Summary

*MuscleX* is a comprehensive open-source data reduction software package for X-ray fiber diffraction patterns from striated muscle and other fibrous systems. The software can be installed on a user’s computer using pre-packaged installers, Python package managers such as *pip* and *conda*, or directly from the source code. It is supported on multiple operating systems including Linux, Microsoft Windows and macOS. The software provides a user-friendly GUI and can be run from the command line for higher throughput or as part of beamline control software data analysis pipelines. Modules are provided for quadrant folding and background subtraction, analysis of equatorial patterns, analysis of the meridian and layer lines, as well as utilities for summing images in various ways to increase the SNR. There is also a simple *X-ray Viewer* module for exploring sequences of images from time-resolved experiments. The suite is under continuous development and new features will continue to be added as requested by the community.

## Data Availability

The *MuscleX* source code is open source under a modified MIT license (‘the IIT license’) and is available at Github (https://github.com/biocatiit/musclex). Documentation for all routines is available at https://github.com/biocatiit/musclex/wiki. Installation packages for Microsoft Windows and MacOS are available online at https://sourceforge.net/projects/musclex/files/latest/download. It is also possible to install either the released or the current development versions of the suite using the *pip* or *conda* Python package installers.
